# Are we near to the end of the standard dose of micafungin?

**DOI:** 10.1186/s13054-018-2068-z

**Published:** 2018-06-05

**Authors:** Alexander Agrifoglio, Lucía Cachafeiro, Eva Herrero, Manuel Sánchez, Abelardo García de Lorenzo

**Affiliations:** Department of Intensive Care Medicine, Hospital Universitario La Paz, Paseo de la Castellana 264, 28046 Madrid, Spain

**Keywords:** Micafungin, Pharmacokinetic, *Candida*

We have carefully read the study titled “Population pharmacokinetics/pharmacodynamics of micafungin against *Candida* species in obese, critically ill, and morbidly obese critically ill patients” [1] and congratulate the authors for such an interesting initiative.

The researchers conclude the lack of adequate micafungin exposure with a 100 mg/24 h dose regardless of the *Candida* species or the patient’s weight. Further, micafungin exposure was adequate to cover *Candida albicans* with a 150 mg/24 h dose for patients weighing up to 115 kg and with a 200 mg/24 h dose for those surpassing this weight. The 200 mg/24 h dose covered *Candida glabrata* for patients weighing up to 115 kg.

These results could correlate with, and also support those that we previously obtained in the first study [2] published on the pharmacokinetics (PK) of micafungin in plasma and burn eschar tissue in critically ill patients with severe burn injuries, which were compared with the PK of micafungin in patients with intra-abdominal infections [3]. In our study, 15 burn patients were compared with ten patients with intra-abdominal infection; all patients were treated with 100 to 150 mg/day of micafungin.

We also observed that the standard dose of micafungin, 100 mg/day, achieves optimal PK/pharmacodynamics (PD) targets in plasma for MIC values of 0.008 mg/L and 0.064 mg/L for non-*parapsilosis Candida* spp. and *Candida parapsilosis*, respectively. By increasing the dose to 200 mg/day, the optimal PK/PD targets in plasma could be achieved for MIC cutoff values that are twofold higher (0.016 mg/L and 0.125 mg/L, respectively).

To these subpopulations of critically ill patients we must add patients with sepsis and mechanical ventilation [4]. The authors recommended dose of 100 mg/day of micafungin would be associated with a very low probability of reaching the AUC_0-24_/MIC ratio in cases of infection with *C. albicans* or *C. glabrata* with MIC ≥ 0.015 mg/L, as well as in almost all cases of infection due to *C. parapsilosis.*

Finally, the conclusions presented above in relation to the most recent PK studies of micafungin, performed in different subpopulations of critically ill patients, would provide us with significant evidence that we should consider an increase in the standard dose (100 mg/day) for the treatment of invasive candidiasis and that it would be advisable, in our opinion, to propose PK/PD studies to patients in whom a lack of clinical or microbiological efficacy due to a suboptimal dose of treatment is suspected.
